# Research on length deformation control of high-altitude linear engineering based on the ellipsoid expansion method

**DOI:** 10.1038/s41598-026-56049-9

**Published:** 2026-06-01

**Authors:** Weixing Yang, Jingjing He, Xinchao Ding, Fangping Li, Li Qian, Lei Gan, Kuangmin Wei

**Affiliations:** 1 POWERCHINA Northwest Engineering Corporation Limited, Xi’an, 710100 China; 2 State Energy Dadu River Jinchuan Hydropower Construction Co., Ltd, Jinchuan, 624100 China; 3 College of Water Resources and Hydropower, Sichuan University, Chengdu, 610065 China; 4 College of Water Conservancy and Hydropower Engineering, Hohai University, Nanjing, 210024 China; 5 Geotechnical Engineering Department, Nanjing Hydraulic Research Institute, Nanjing, 210024 China

**Keywords:** Engineering projection, Length distortion, Ellipsoid expansion, Engineering-optimized ellipsoid, Generalized differential method, Engineering, Mathematics and computing

## Abstract

In engineering control surveys, the problem of length distortion arising from the reduction of field-measured distances to the Gauss plane has long posed challenges in practical applications, particularly in high-altitude linear engineering projects where such distortions are more pronounced. Effective methods for controlling this distortion remain unclear. This paper systematically analyzes the deformation patterns of ground survey distances reduced to the ellipsoid and then projected onto the Gauss plane. It also evaluates and compares the effectiveness of four ellipsoid expansion methods—mean curvature radius method, prime vertical curvature radius method, planar analytical method, and generalized differential calculus method—in establishing engineering ellipsoids for distortion control. The results demonstrate that the engineering ellipsoid constructed via the generalized differential calculus method achieves optimal control over length distortion, with a maximum distortion of only 11.4 mm, well below the specified tolerance limit (± 37.9 mm). The findings confirm that this method provides a reliable technical basis for establishing coordinate systems in linear engineering projects located in high-altitude regions.

## Introduction

In modern high-precision engineering construction, accurate length measurement serves as the foundation for ensuring project quality and safety^1^. However, significant length distortions are often introduced when ground-measured lengths are successively projected onto the reference ellipsoid and the Gaussian plane^2^. This issue is particularly pronounced in long-distance linear engineering projects, such as high-speed railways, large-scale hydraulic hubs, and long-distance pipelines^3–6^. If the resulting length distortion exceeds the standard limits (e.g., 25 mm/km), it can severely compromise design accuracy, construction positioning precision, and even operational safety^7–9^. Therefore, effectively controlling and eliminating length distortion during the measurement process to achieve a high degree of consistency between plan lengths and field lengths has become a critical technical challenge in contemporary engineering surveying^3,10,11^.

Therefore, this study proposes the adoption of the ellipsoid expansion method to construct an engineering reference ellipsoid that achieves an optimal fit with the project area^12–14^. By adjusting the ellipsoidal parameters, this method ensures that the ellipsoidal surface approximately coincides with or maintains a fixed relationship to the average elevation surface of the surveyed area^15,16^. This approach effectively mitigates length distortions resulting from the two-step projection process at the source, thereby strictly controlling the deformation within the limits specified by relevant standards^3,17,18^. As a result, high-fidelity length transfer from the field to design drawings is achieved.

To validate the effectiveness of the proposed method, this study systematically investigates four typical ellipsoid expansion modeling approaches: the mean curvature radius method, the prime vertical curvature radius method, the planar analytical method, and the generalized differential method^3,14,18,19^. Through theoretical derivation, numerical simulation, and comparative analysis of measured data, a comprehensive evaluation is conducted to assess the applicability and accuracy performance of each method under different topographic conditions and across various latitude ranges, thereby determining the optimal modeling strategy^20–22^.

The findings of this study demonstrate that among various ellipsoid expansion methods, the generalized differential method exhibits significant advantages in constructing engineering reference ellipsoids^23^. With its rigorous mathematical structure, this method enables flexible and precise determination of ellipsoid expansion parameters through a generalized differential model, thereby achieving an optimal fit between the engineering ellipsoidal surface and the average elevation surface of the surveyed area^3,4,24,25^. Final calculations confirm that the engineering ellipsoid established based on this method effectively minimizes projection distortion to the greatest extent, providing a reliable technical pathway for resolving the issue of length distortion.

The systematic comparative results demonstrate that the generalized differential ellipsoidal expansion method possesses a core advantage in controlling projection distortion. The engineering ellipsoid model established using this method provides critical support for high-precision measurement control networks. The application of this achievement not only directly ensures engineering geometric quality and spatial reference accuracy but also promotes further refinement of the technical framework for precision engineering surveying.

## Materials and methods

### Study area

**Fig. 1:**
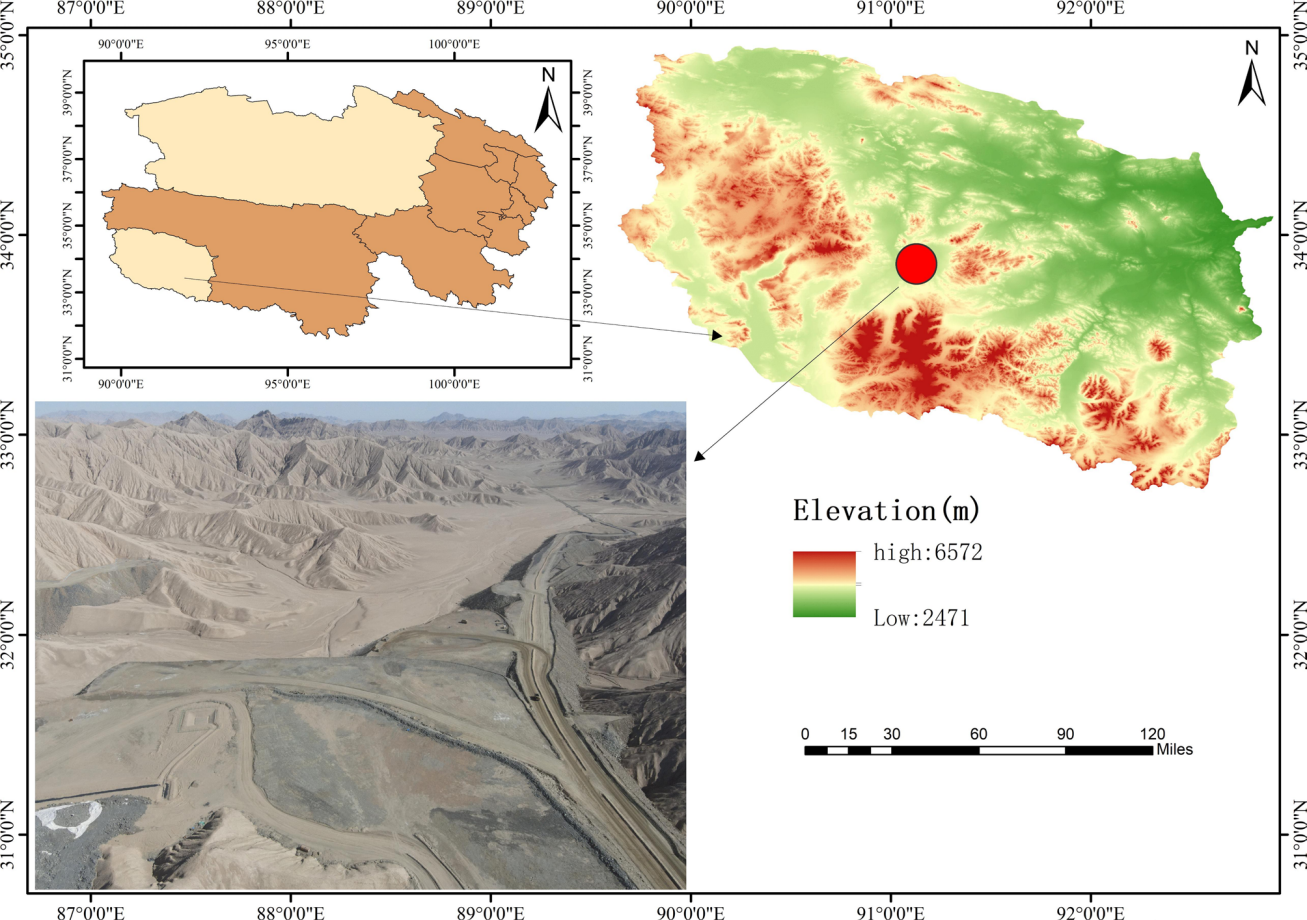
Location of the Xiarihamu Ni–Co deposit. The map consists of three panels: the upper left shows the administrative divisions of Qinghai Province; the upper right presents a digital elevation model (DEM) of the Golmud region, with the red dot indicating the location of the Xiarihamu Ni–Co deposit; and the lower left displays a remote sensing image of the deposit. This figure was originally created by myself using the open-source software QGIS (version 3.40.7, URL: https://www.gnu.org).

The Xiarihamu nickel-cobalt deposit is located on the southern margin of the Qaidam Basin, in the western segment of the East Kunlun Mountains, near Golmud City, Qinghai Province. Situated at an average elevation of approximately 3,600 m, it lies within a high-altitude transitional zone between the Kunlun Mountains and the Qaidam Basin. Administratively, it belongs to the Wutumeiren Township, with the Nalinggele River flowing through the area. The open-pit mine has a maximum mining level of 3695 m and a minimum of 3020 m, resulting in a maximum slope height of 675 m (Fig. 1). The study area is centered at 35.58°N, with a central meridian of 111° in the 6° longitudinal zone, an ellipsoidal height of 3437 m, and an east–west extent of 50 km.

### Experimental design

**Fig. 2:**
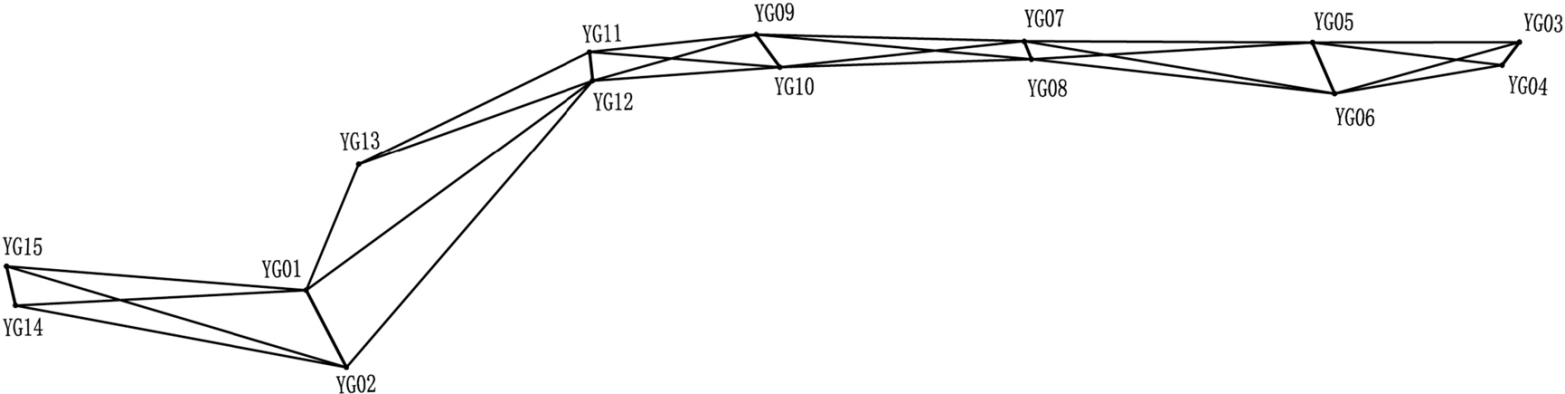
Diagram of the survey control network distribution in the mining area.

In this study, a set of 15 GNSS control points was established along a mining area belt line to obtain geodetic coordinates (longitude, latitude) and ellipsoidal heights (Fig. 2). The GNSS static survey was carried out in compliance with Class C control network specifications. Seven Leica GNSS receivers were used for synchronous observation, with the instrumental fixed error less than 3 mm and proportional error less than 3 mm. This study adopts the China Geodetic Coordinate System 2000 (CGCS2000), with major semi-axis * a* = 6,378,137 m and flattening * f* = 1/298.257222101.

First, the height of the engineering compensation surface for the study area was calculated and established, and the measured ground lengths were reduced to this surface. Subsequently, an engineering ellipsoid was constructed based on the ellipsoid expansion principle, and the ground measurements were further reduced to this ellipsoidal surface.

To evaluate the reduction accuracy, the actual distances of seven selected edges were measured using a TS60 measurement robot as reference values. Field observations were performed using the Leica TS60 total station with both face-left and face-right measurements for a total of 12 observation sets. In prism measurement mode, its nominal ranging accuracy is 0.6 mm + 1 ppm and angular measurement accuracy is 0.5 arcseconds. Atmospheric correction was automatically implemented based on on-site environmental parameters. By comparing the projection distortions of ground lengths reduced to the compensation surface and the engineered ellipsoid surface, an optimal engineering projection surface was identified.

### Index and method

#### Reduction and distortion of terrestrial measured distances to the projection plane

**Fig. 3:**
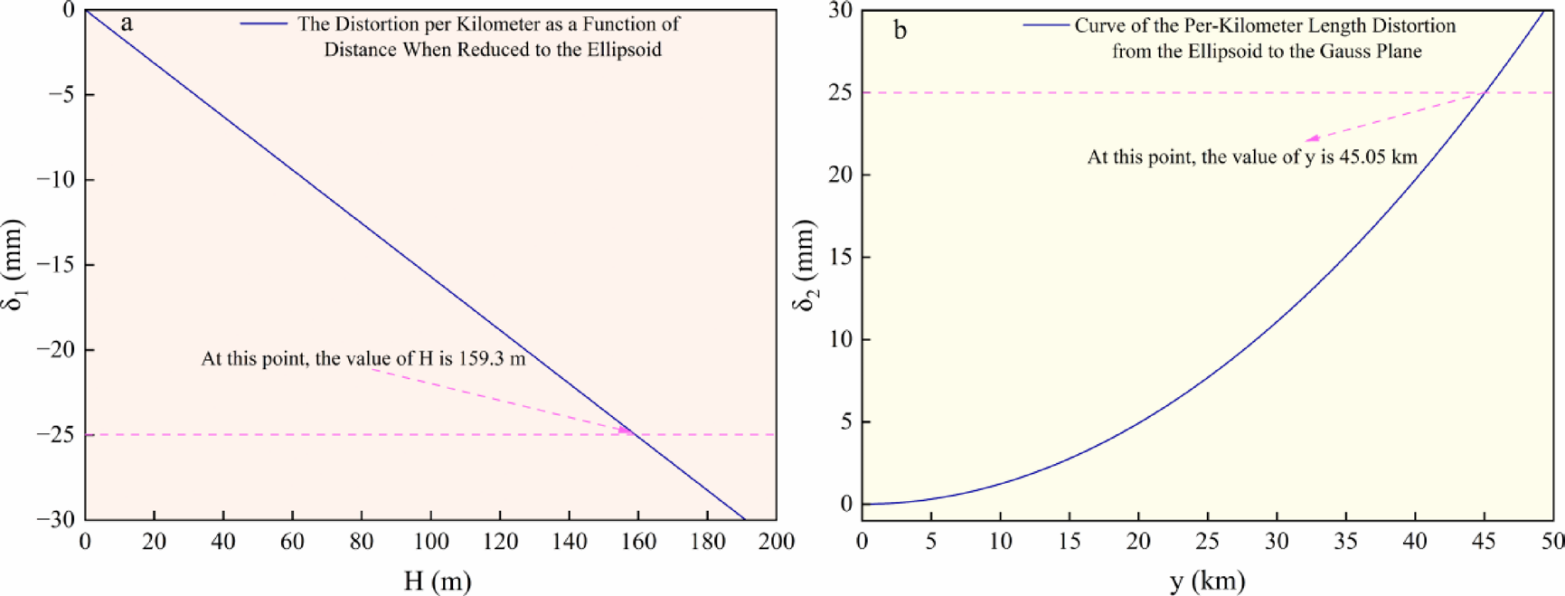
Per-kilometer length changes from projecting ground distances onto different reference surfaces. (**a**) Reduction to ellipsoid: the change $\:{\delta\:}_{1}$ reaches 25 mm at an average elevation (H) of 159.3 m. (**b**) Projection to Gauss plane: the change $\:{\delta\:}_{2}$ reaches 25 mm at a y-coordinate of 45.05 km.

In engineering surveying, the measured ground distances must be rigorously reduced to the Gaussian projection plane to ensure a unified coordinate system and practical consistency. This process involves two critical stages: first, the ground-level observed distances are reduced to the reference ellipsoid, which generally results in a shortening deformation; subsequently, these ellipsoidal distances are projected onto the Gaussian plane, typically leading to a lengthening deformation (Fig. 3). To meet engineering accuracy standards, the resultant length distortion after projection must generally be controlled within 25 mm/km. The specific reduction formulas are as follows:

1 $$\:{S}_{1}=(1-\frac{H}{R}){S}_{0}$$

where we denote by $\:{S}_{0}$ the measured horizontal distance on the ground, by $\:{S}_{1}$ the distance after reduction to the reference ellipsoid, by * H* the average elevation of the segment endpoints, and by * R* the mean radius of curvature of the ellipsoid in the survey area, which is conventionally set to 6371 km. Therefore, based on Eq. (1), the deformation per kilometer, $\:{\delta\:}_{1}$ (Fig. 3a), resulting from the reduction of a ground-surveyed length to the reference ellipsoid can be derived as:

2 $$\:{\delta\:}_{1}=-\frac{H}{R}$$

Simultaneously, when the length $\:{S}_{1}$ on the ellipsoid is transformed to the Gauss plane through Gauss projection, the calculation formula for the corresponding length $\:{S}_{2}$ is:

3 $$\:{S}_{2}=(1+\frac{{y}^{2}}{2{R}^{2}}){S}_{1}$$

where we denote by $\:{S}_{1}$ the length on the ellipsoid, by $\:{S}_{2}$ its projected length on the Gauss plane, and by * y* the mean horizontal coordinate of the endpoints on that plane. This formulation is simplified, as we have omitted all higher-order small terms.

Thus, from Eq. (3) above, the expression for the per-kilometer length deformation $\:{\delta\:}_{2}$ (Fig. 3b), resulting from the projection of the ellipsoidal length onto the Gauss plane, can be derived as follows:

4 $$\:{\delta\:}_{2}=\frac{{y}^{2}}{2{R}^{2}}$$

where $\:{\delta\:}_{2}$ represents the deformation per kilometer for an ellipsoidal length projected onto the Gauss plane.

**Fig. 4:**
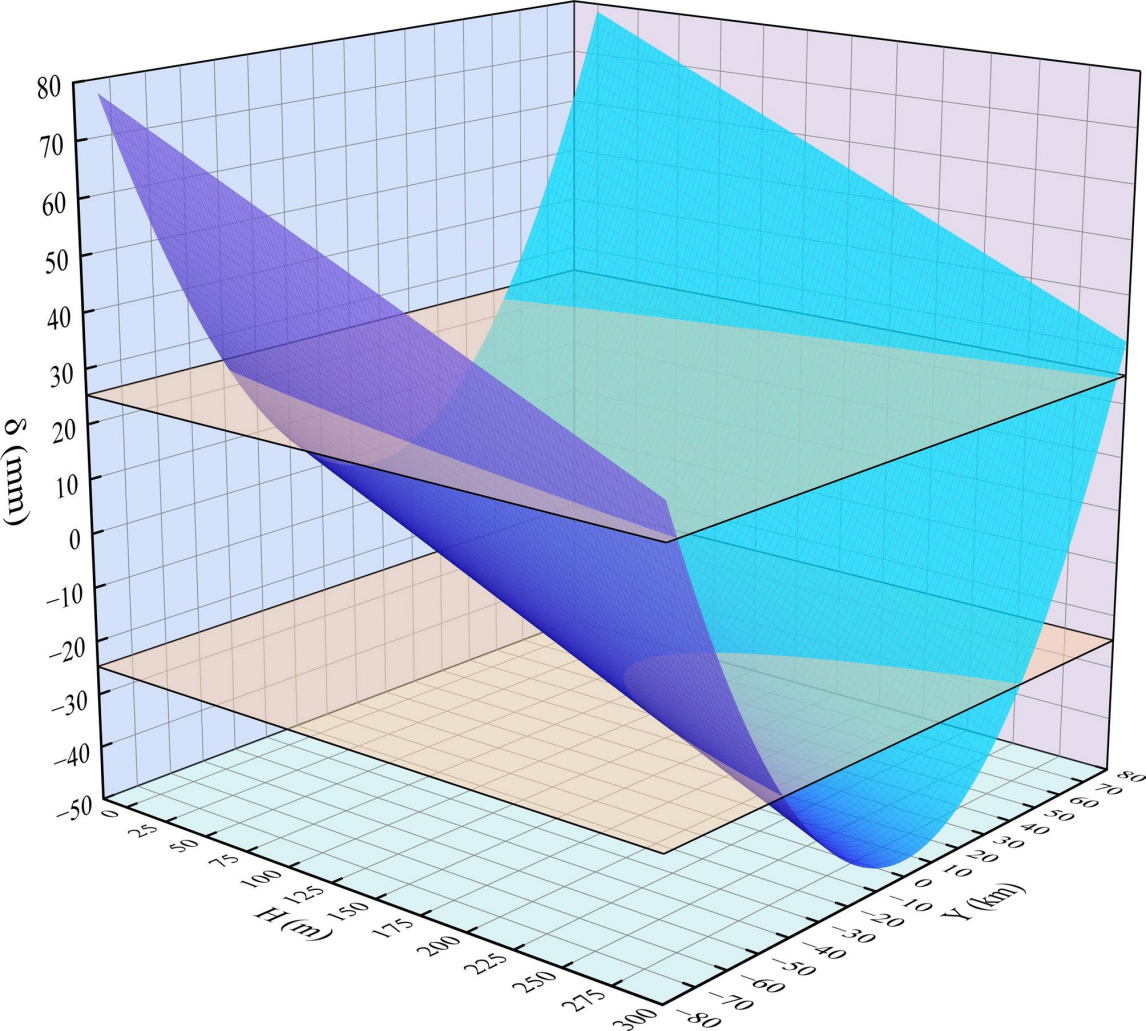
Three-dimensional deformation surface for the projection of ground-surveyed lengths to the Gauss plane. The colored surface region located between the δ = 25 mm and δ = -25 mm horizontal planes corresponds to the condition where the total deformation per kilometer is within the permissible limit of 25 mm for engineering surveys.

Therefore, by combining Eqs. (2) and (4), the formula for the combined deformation $\:\delta\:$ (Fig. 4), resulting from the projection of a ground-surveyed length onto the Gauss plane, can be derived as follows:

5 $$\:\delta\:=-\frac{H}{R}+\frac{{y}^{2}}{2{R}^{2}}$$

Equation (5) and Fig. 4 jointly illustrate the response mechanism of the combined deformation $\:\delta\:$ when ground-surveyed lengths are projected onto the Gauss plane. The value of $\:\delta\:$ is strongly influenced by the elevation * H* and the y-coordinate. The absolute value of $\:\delta\:$ shows a systematic increase with * H*, indicating that elevation is a key driver of significant projection deformation. When H reaches extreme values (e.g., 159.3 m), $\:\left|\delta\:\right|$ peaks at ± 25 mm/km, further confirming the monotonic increasing nature of the elevation effect. Simultaneously, deviation in the y-coordinate also significantly affects the deformation distribution, with $\:\left|\delta\:\right|$ reaching the same critical value in extreme regions (e.g., * y* ≈ ±45.05 km), consistent with the theoretical expectation that length distortion increases with the y-coordinate in Gauss projection. Consequently, the surface region bounded by the $\:\delta\:$ = ±25 mm planes in Fig. 4 can be regarded as the effective parameter domain satisfying the engineering survey specification of a ± 25 mm/km tolerance.

#### Ellipsoid expansion and datum establishment

The core finding of this study demonstrates that, for linear long-distance engineering projects, the use of the ellipsoidal expansion method to establish an independent project coordinate system serves as an effective approach to controlling projection length distortion. By optimizing the parameters of the reference ellipsoid, this method enables effective control of length distortion across the entire project route, ensuring strict compliance with the engineering specification of no more than 25 mm/km.

The principle of the ellipsoidal expansion method is to adjust the semi-major axis of the reference ellipsoid while keeping the ellipsoid center, flattening and orientation unchanged. This operation aligns the ellipsoidal surface with the mean elevation surface of the survey area, so as to systematically correct Gauss projection distortion fundamentally. In short, the original positioning and orientation parameters remain unchanged. The ellipsoid is scaled with a fixed flattening value, and the adjusted ellipsoidal surface can match the projection datum plane adopted by the independent coordinate system. All four ellipsoid expansion methods adopted in this study are analyzed and calculated on the premise that the flattening of the original ellipsoid remains constant^26^.

During implementation, the optimal adjustment to the semi-major axis is first determined. Based on the new ellipsoid, geodetic coordinates are then inversely calculated, and Gauss projection is finally performed to obtain plane coordinates with controlled distortion. This approach effectively mitigates projection distortions caused by remote locations or high elevations in the survey area, providing a reliable datum for high-precision surveying applications such as long-linear engineering projects.

**Fig. 5:**
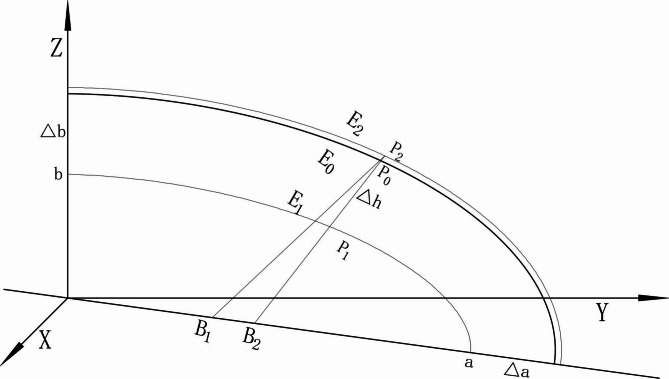
Principle of ellipsoidal expansion for projection distortion control.

As illustrated in Fig. 5, a point $\:{P}_{1}$ on the original ellipsoid $\:{E}_{1}$ is elevated by a height $\:{\Delta\:}h$ along the direction of its prime vertical curvature radius to reach point $\:{P}_{2}$ on the expanded ellipsoid $\:{E}_{2}$, achieving approximate coincidence with the target point $\:{P}_{0}$ on the engineering reference ellipsoid $\:{E}_{0}$.

According to the preceding theoretical analysis, the inflated ellipsoid maintains consistency with the original ellipsoid in terms of flattening, position, and orientation. Consequently, the transformation between the spatial coordinates of any point on the inflated ellipsoid and its corresponding point on the original ellipsoid involves no changes in the coordinate transformation parameters. Specifically, all seven parameters in the transformation model - three translation, three rotation, and one scale parameter - remain zero. This result further demonstrates that the ellipsoid inflation process is geometrically conformal and consistent, making it suitable for the continuous transformation analysis of spatial coordinates. Hence, the change in spatial coordinates for any point after ellipsoid inflation is:

6 $$\:\left[\begin{array}{c}dL\\\:dB\\\:dH\end{array}\right]=\left[\begin{array}{c}0\\\:\frac{N}{(M+H)a}{e}^{2}\mathrm{sin}B\mathrm{cos}B{\rho\:}^{{\prime\:\prime\:}}\\\:-\frac{N}{a}(1-{e}^{2}{sin}^{2}B)\end{array}\right]\left[\varDelta\:a\right]$$

where * dL*, * dB*, * dH*, and * da* are the increments in longitude, latitude, ellipsoidal height, and semi-major axis resulting from the inflation; the parameters of the original ellipsoid are: * a* for the semi-major axis, * B* for the geodetic latitude of the point, and * e* for the first eccentricity; * M* and * N* signify the meridian and prime vertical radii of curvature at the point on the original ellipsoid, respectively; and $\:{\rho\:}^{{\prime\:\prime\:}}$ is the arc-second conversion constant, with a value of 206,265″.

The construction of an engineering ellipsoid via the inflation method is a recognized technique for minimizing projection distortion of distances. The critical task is to compute the variation in the semi-major axis, * da*. Four principal methods exist for this purpose: the mean radius of curvature, the prime vertical radius of curvature, the planar analytical, and the generalized differential methods. It is recognized that the planar analytical and generalized differential methods provide greater theoretical robustness and accuracy, and are therefore recommended for applications demanding high precision.

##### Mean radius of curvature method

The Mean Radius of Curvature Method is designed to determine the incremental change in the semi-major axis, * da*, by inflating the ellipsoid along the direction of the mean radius of curvature at the central point of the survey area until it reaches the projection plane height. The derivation rests on two key premises: the geodetic latitude of the central point remains unchanged before and after inflation, and higher-order infinitesimal terms are neglected. Under these premises, the formula for * da* is derived as follows:

7 $$\:da=\frac{1-{e}^{2}{sin}^{2}B}{\sqrt{1-{e}^{2}}}\varDelta\:H$$

where $\:\varDelta\:H$ is defined as the ellipsoidal height of the projection plane, representing its vertical separation from the surface of the original ellipsoid.

##### Prime vertical radius of curvature method

The core of this method involves inflating the ellipsoid along the normal direction at the central point of the survey area. The derivation relies on two key assumptions: first, that the geodetic latitude of the central point remains unchanged before and after the inflation; and second, that the increment of the prime vertical radius of curvature at this point equals the ellipsoidal height of the projection surface, $\:\varDelta\:H$. Consequently, the formula for the semi-major axis change, * da*, is derived as follows:

8 $$\:da=\varDelta\:H\sqrt{1-{e}^{2}{sin}^{2}B}$$

##### Planar analytical method

The planar analytical method improves upon the mean and prime vertical radius methods by accounting for variations in geodetic latitude and normal deflection during inflation. Consequently, it delivers a more rigorous and accurate solution for the semi-major axis change, * da*, as expressed by the following formula:

9 $$\:{B}_{1}={\mathrm{tan}}^{-1}\left[(1+\frac{\varDelta\:H{e}^{2}}{(N+\varDelta\:H)(1-{e}^{2})})\mathrm{tan}B\right]$$

10 $$\:{N}_{1}=\frac{(N+\varDelta\:H)\mathrm{cos}B}{\mathrm{cos}{B}_{1}}$$

11 $$\:da={N}_{1}\sqrt{1-{e}^{2}{sin}^{2}{B}_{1}}-a$$

where * a* is the semi-major axis of the original ellipsoid. For the central point of the survey area, * B* and * N* are its geodetic latitude and prime vertical radius of curvature on the original ellipsoid, while $\:{B}_{1}$ and $\:{N}_{1}$ are the same geometric parameters on the inflated ellipsoid.

##### Generalized differential method

The generalized differential method offers a more rigorous theoretical framework than the planar analytical method. It first systematically derives the coordinate variations (latitude, longitude, and ellipsoidal height) of a point after ellipsoid inflation based on the generalized geodetic coordinate differential formula, as shown in Eq. (6). This establishes a rigorous foundation for subsequent calculations. Subsequently, by relating the variation in ellipsoidal height to the formula for the prime vertical radius of curvature, the formula for the semi-major axis change, * da*, is ultimately obtained:

12 $$\:da=\frac{\varDelta\:H}{\sqrt{1-{e}^{2}{sin}^{2}B}}$$

## Results

### Variations in the semi-major axis by different ellipsoid expansion methods

**Fig. 6:**
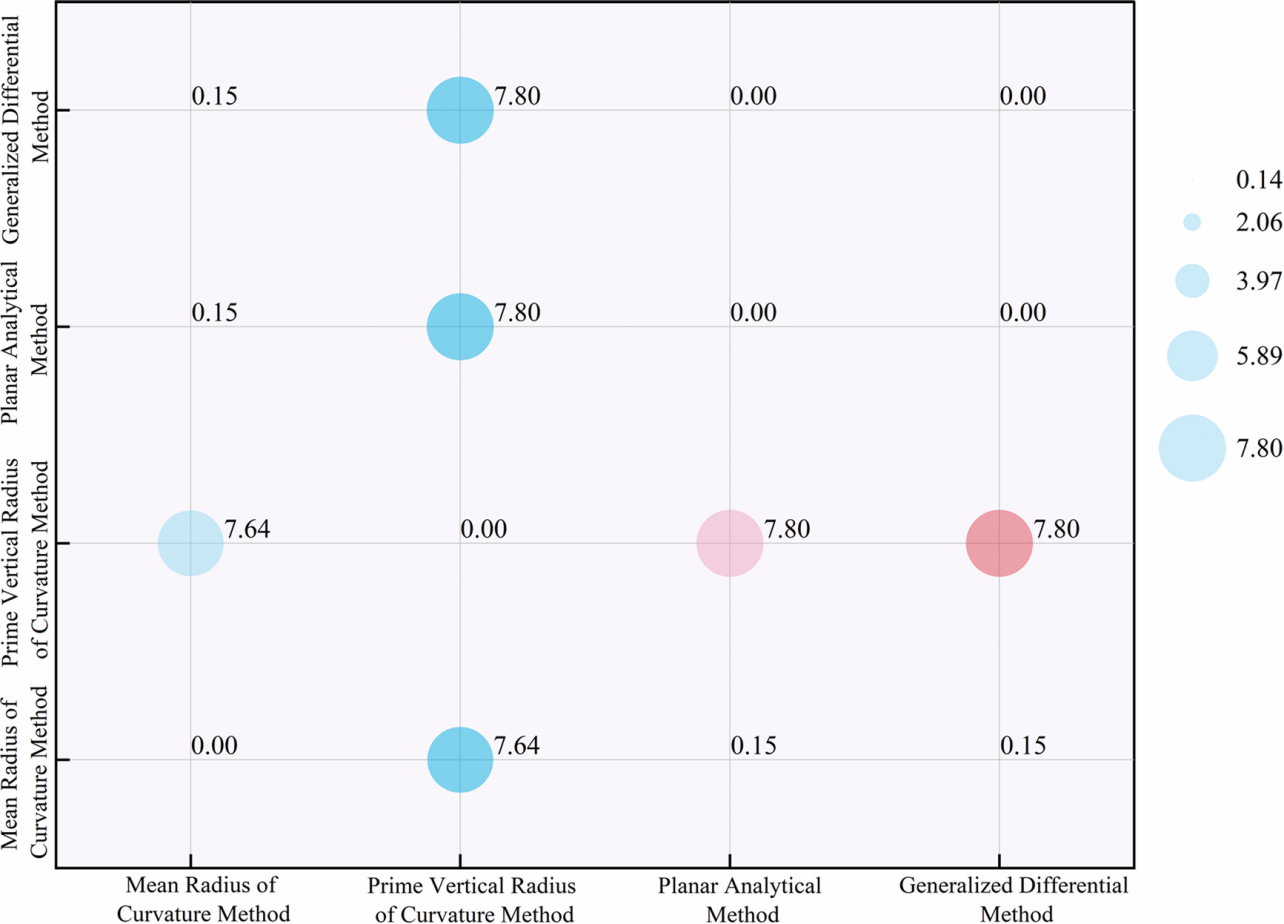
Pairwise comparison matrix of semi-major axis changes resulting from various ellipsoid expansion methods. Each bubble, positioned at the matrix intersection, is scaled in proportion to the absolute difference in the change values between the two respective methods.

The four ellipsoid expansion methods all significantly affected the semi-major axis of the ellipsoid at the center of the survey area. Among them, the result from the Prime Vertical Radius of Curvature Method was lower than the original ellipsoidal geodetic height of 3437 m, while the results from the other three methods were all higher than this value (Table 1). In cross-comparisons among the methods, the Mean Radius of Curvature Method showed obvious differences compared to the other three methods, with a maximum difference of 7.8 m (Table 1; Fig. 6). Similarly, the Prime Vertical Radius of Curvature Method also exhibited obvious differences relative to the other three methods. In contrast, the difference between the Planar Analytical Method and the Generalized Differential Method was small differences, being less than 0.01 mm (Table 1; Fig. 6).

**Table 1:** Semi-major axis variation from ellipsoidal expansion by different models (m).

Mean radius of curvature method	Prime vertical radius of curvature method	Planar analytical method	Generalized differential method
3440.746922	3433.103165	3440.901267	3440.901258

### Variations in latitude and geodetic height at various points induced by different ellipsoid expansion methods

**Fig. 7:**
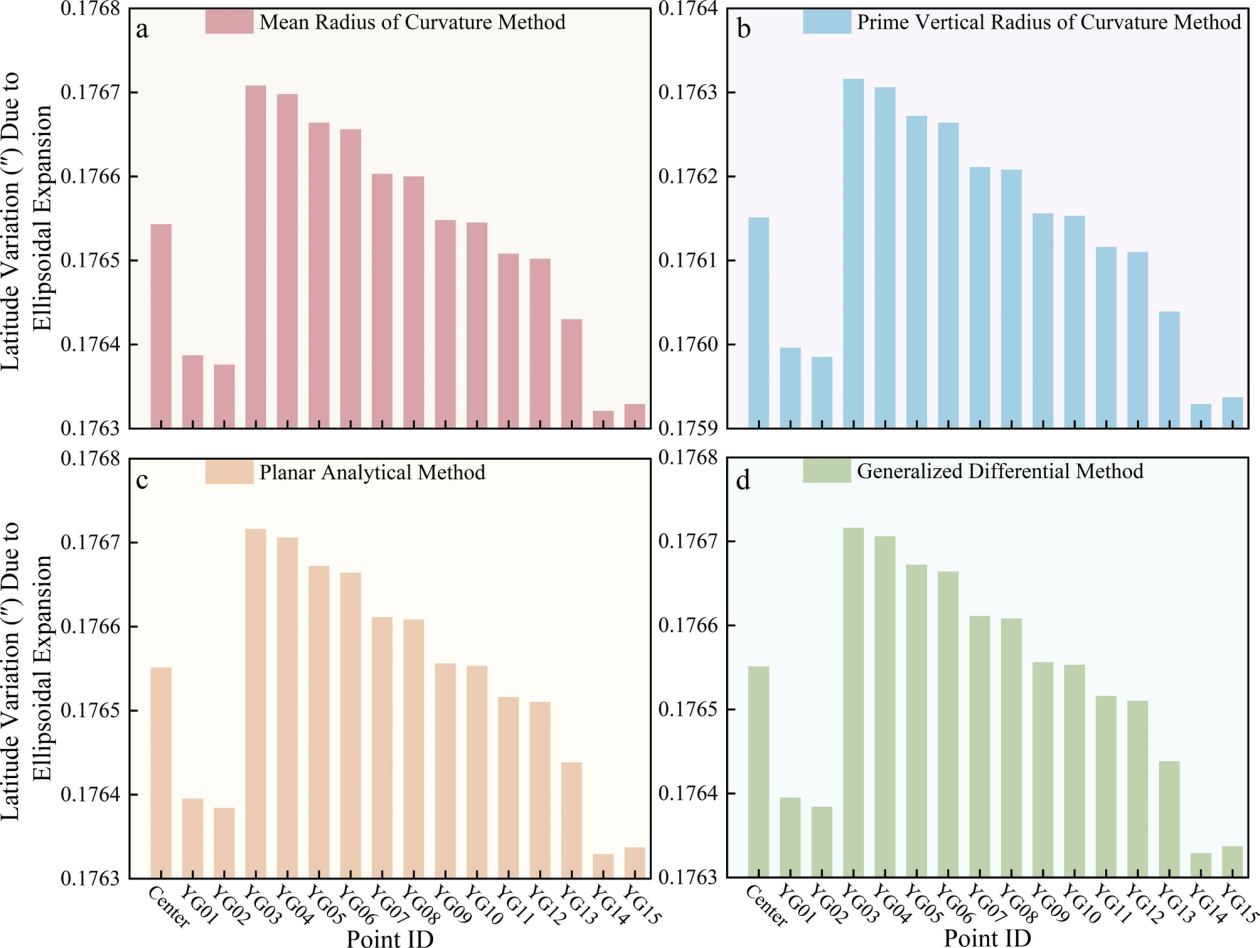
Model comparisons of latitude variations induced by ellipsoidal expansion. Subplots (**a**)–(**d**) correspond to four computational models: the Mean Radius of Curvature Method, the Prime Vertical Radius of Curvature Method, the Planar Analytical Method, and the Generalized Differential Method, respectively.

Under different models, the ellipsoid expansion induces certain variations in the latitudes of both the center point and the sampling points (Table 2; Fig. 7). Specifically, the Mean Radius of Curvature Method yields the smallest range of latitude variation, between 0.176321″ and 0.176708″, with a mean value of 0.176526″. The Prime Vertical Radius of Curvature Method results in a variation range of [0.175929″, 0.176316″], with a mean of 0.176134″. Both the Planar Analytical Method and the Generalized Differential Method exhibit identical variation ranges of [0.176329″, 0.176716″], with mean values of 0.176534″. Overall, the latitude variations caused by all models are relatively small, with the full fluctuation interval being [0.175929″, 0.176716″] (Table 2). Furthermore, under all four models, the influence of ellipsoid expansion on latitude exhibits a proportional relationship with the point distance, and the trends of variation are generally consistent. It is particularly noteworthy that the Planar Analytical Method and the Generalized Differential Method produce nearly identical trends in latitude variation for both the center and all sampling points.

**Table 2:** Changes in latitude and ellipsoidal height at various points due to ellipsoidal expansion under different models.

Point ID	Mean radius of curvature method		Prime vertical radius of curvature method		Planar analytical method		Generalized differential method	
	Latitude (″)	Ellipsoidal height (m)	Latitude (″)	Ellipsoidal height (m)	Latitude (″)	Ellipsoidal height (m)	Latitude (″)	Ellipsoidal height (m)
Center	0.176543	0.154161	0.176151	7.789251	0.176551	-0.000009	0.176551	0.000000
YG01	0.176387	0.140017	0.175996	7.775138	0.176395	-0.014154	0.176395	-0.014145
YG02	0.176376	0.139035	0.175985	7.774158	0.176384	-0.015137	0.176384	-0.015127
YG03	0.176708	0.169208	0.176316	7.804265	0.176716	0.015038	0.176716	0.015048
YG04	0.176698	0.168345	0.176306	7.803403	0.176706	0.014175	0.176706	0.014184
YG05	0.176664	0.165198	0.176272	7.800264	0.176672	0.011028	0.176672	0.011038
YG06	0.176656	0.164454	0.176264	7.799522	0.176664	0.010284	0.176664	0.010294
YG07	0.176603	0.159641	0.176211	7.794719	0.176611	0.005471	0.176611	0.005480
YG08	0.176600	0.159363	0.176208	7.794441	0.176608	0.005192	0.176608	0.005202
YG09	0.176548	0.154589	0.176156	7.789678	0.176556	0.000419	0.176556	0.000428
YG10	0.176545	0.154299	0.176153	7.789389	0.176553	0.000129	0.176553	0.000138
YG11	0.176508	0.150974	0.176116	7.786072	0.176516	-0.003196	0.176516	-0.003187
YG12	0.176502	0.150376	0.176110	7.785474	0.176510	-0.003795	0.176510	-0.003786
YG13	0.176430	0.143912	0.176039	7.779024	0.176438	-0.010259	0.176438	-0.010250
YG14	0.176321	0.134036	0.175929	7.769171	0.176329	-0.020136	0.176329	-0.020126
YG15	0.176329	0.134754	0.175937	7.769887	0.176337	-0.019418	0.176337	-0.019408

**Fig. 8:**
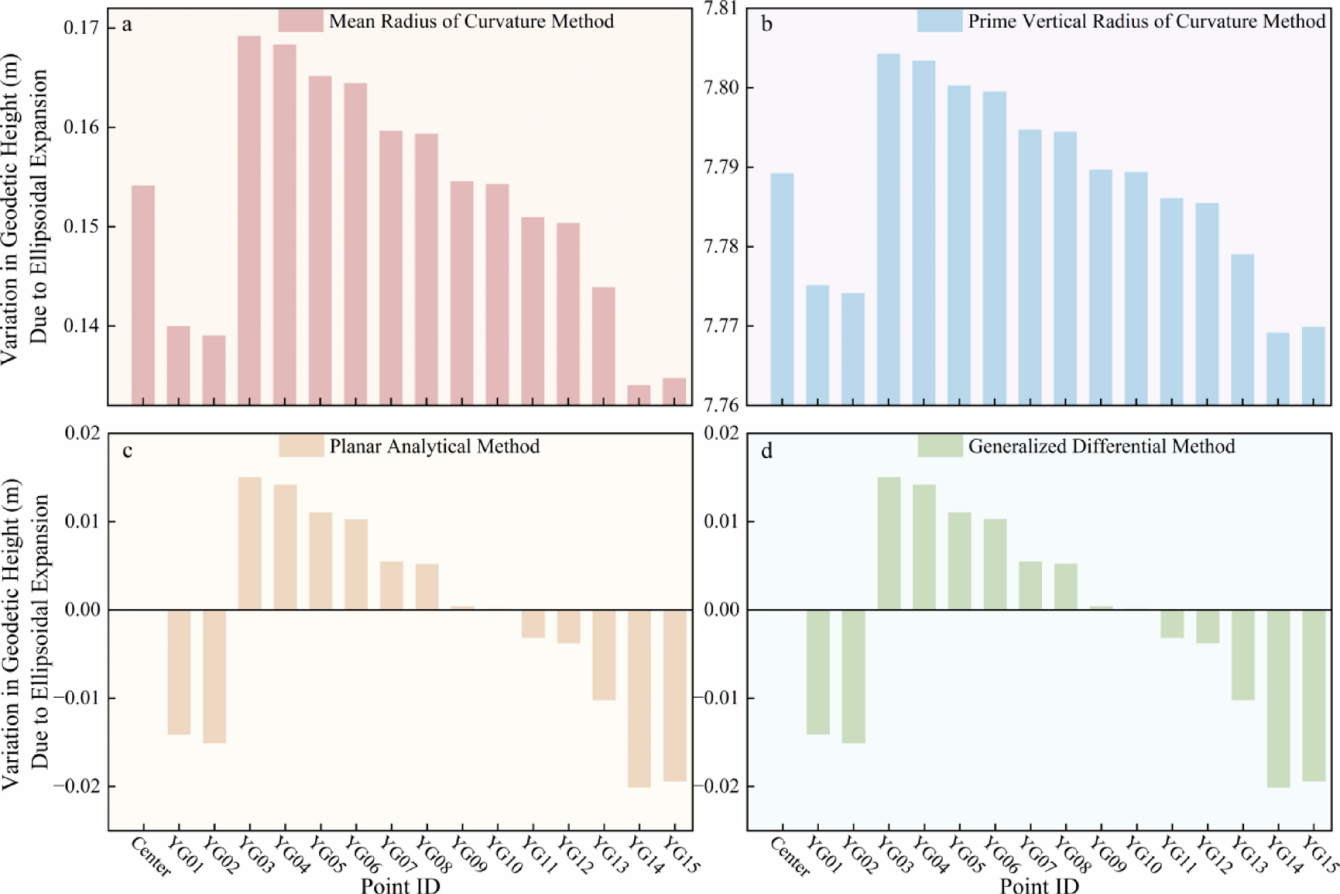
Comparison of calculated geodetic height changes induced by ellipsoidal expansion from different models. Subplots (**a**)–(**d**) correspond to four computational models: the mean radius of curvature method, the prime vertical radius of curvature method, the planar analytical method, and the generalized differential method, respectively.

In contrast to the latitude variations, the ellipsoidal expansion induced more pronounced changes in ellipsoidal height across the different models. All four models resulted in varying degrees of alteration in the ellipsoidal height at both the center point and the sampling points. Specifically: The Mean Radius of Curvature Method produced height changes ranging from 0.134036 m to 0.169208 m, with a mean value of 0.152648 m. The Prime Vertical Radius of Curvature Method resulted in a variation between 7.769171 m and 7.804265 m, averaging 7.787741 m. The Planar Analytical Method led to height differences from − 0.020136 m to 0.015038 m, with a mean of − 0.001523 m. The Generalized Differential Method showed variations from − 0.020126 m to 0.015048 m, and a mean value of − 0.001514 m (Table 2; Fig. 8).

Notably, the ellipsoidal height variation caused by the Prime Vertical Radius of Curvature Method was significantly larger than those of the other models, reaching approximately 7.8 m. The Mean Radius of Curvature Method resulted in the second largest variation, around 0.15 m, whereas both the Planar Analytical Method and Generalized Differential Method induced only millimeter-level changes (Table 2; Fig. 8). In particular, the ellipsoidal height change at the center point was nearly zero under the Generalized Differential Method, indicating that this method achieves the best consistency with the projected height after ellipsoidal expansion.

### Analysis of length distortion in different map projections

In this study, the lengths of seven baselines within the survey area were measured using a high-precision TS60 measurement robot. These measured lengths were compared with their counterparts reduced to the 3437 m engineering projection plane and those projected onto an engineering ellipsoid expanded via the generalized differential ellipsoid expansion method.

**Fig. 9:**
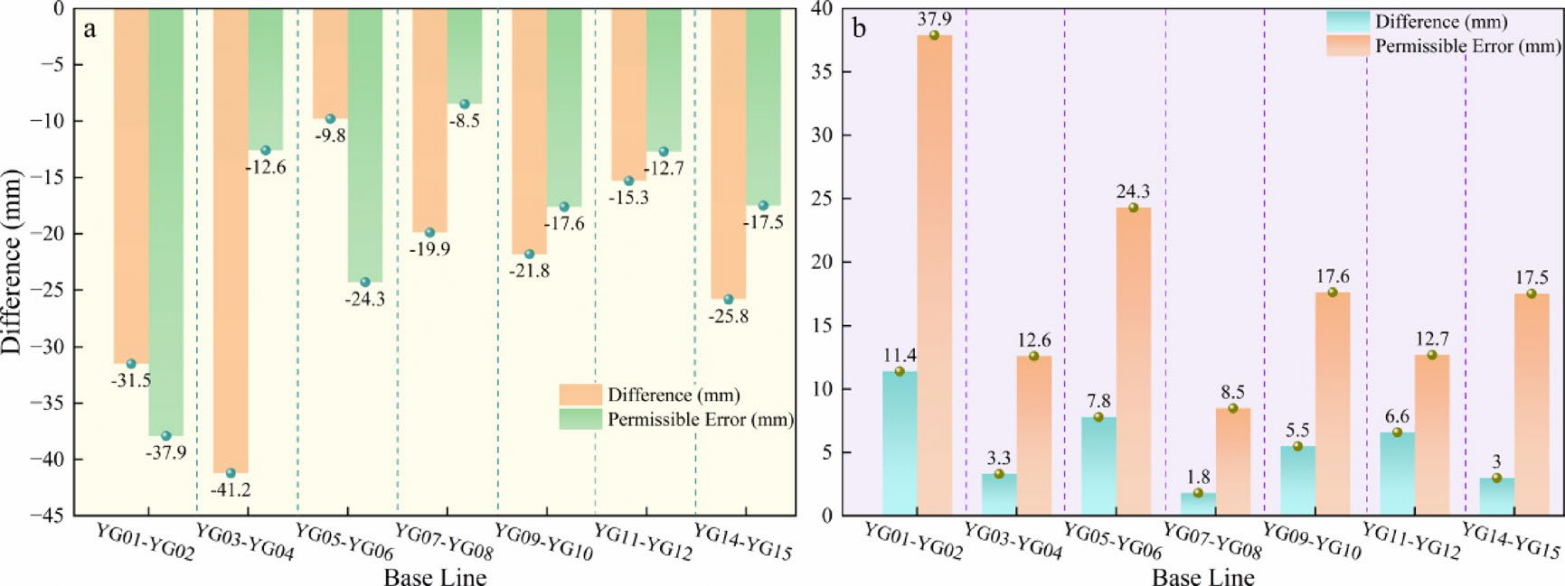
Comparison of length discrepancies from EDM measurements projected onto different coordinate surfaces: (**a**) the engineering projection plane and (**b**) the engineering ellipsoidal surface.

When the baselines were reduced to the 3437 m engineering projection plane, the length distortions of five out of the seven baselines exceeded the tolerance limits specified by the engineering standards, with the exception of baselines YG01–YG02 and YG05–YG06 (Table 3; Fig. 9a). In contrast, projection onto the engineering ellipsoid after generalized differential expansion resulted in length distortions for all baselines that remained within the permissible limits and were substantially lower than the threshold values (Table 3; Fig. 9b).

**Table 3:** Comparison of electronic distance measurement (EDM) measurements with distances reduced to the engineering projection plane.

Base line	Electro-optical distance (m)	Projection plane distance (m)	Difference (mm)	Permissible error (mm)
YG01–YG02	1517.7516	1517.7831	− 31.5	± 37.9
YG03–YG04	503.2254	503.2666	− 41.2	± 12.6
YG05–YG06	972.3593	972.3691	− 9.8	± 24.3
YG07–YG08	341.4682	341.4881	− 19.9	± 8.5
YG09–YG10	704.0908	704.1126	− 21.8	± 17.6
YG11–YG12	506.1111	506.1264	− 15.3	± 12.7
YG14–YG15	700.2534	700.2792	− 25.8	± 17.5

**Table 4:** A comparison between electro-optical measurements and engineering ellipsoid distances.

Base line	Electro-optical distance (m)	Engineering Ellipsoid distance (m)	Difference (mm)	Permissible error (mm)
YG01-YG02	1517.7516	1517.7402	11.4	± 37.9
YG03-YG04	503.2254	503.2221	3.3	± 12.6
YG05-YG06	972.3593	972.3515	7.8	± 24.3
YG07-YG08	341.4682	341.4664	1.8	± 8.5
YG09-YG10	704.0908	704.0853	5.5	± 17.6
YG11-YG12	506.1111	506.1045	6.6	± 12.7
YG14-YG15	700.2534	700.2504	3.0	± 17.5

**Table 5:** Difference in length between electro-optical measurements and different ellipsoid expansion methods.

Base line	Difference in length between electro-optical measurements and different ellipsoid expansion methods (mm)					Permissible error (mm)
	Electro-optical distance (m)	Mean radius of curvature method	Prime vertical radius of curvature method	Planar analytical method	Generalized differential method	
YG01–YG02	1517.7516	35.8	28.5	13.7	11.4	± 37.9
YG03–YG04	503.2254	11.1	12.7	4.8	3.3	± 12.6
YG05–YG06	972.3593	24.6	19.1	12.4	7.8	± 24.3
YG07–YG08	341.4682	5.4	5.3	2.6	1.8	± 8.5
YG09–YG10	704.0908	16.8	11.4	7.3	5.5	± 17.6
YG11–YG12	506.1111	20.5	17.5	9.4	6.6	± 12.7
YG14–YG15	700.2534	9.7	8.7	4.6	3.0	± 17.5

## Discussion

This study aims to systematically address a key issue in precision engineering surveying: how to project ground-based electro-optical distance measurements onto an engineering projection plane in the most reasonable, accurate, and faithful manner. The core of achieving this goal lies in establishing a custom engineering ellipsoid that closely fits the regional engineering average elevation surface in the survey area^16,27^. To this end, four mainstream ellipsoid expansion methods were systematically compared in order to identify the optimal mathematical model and implementation strategy for this crucial step. The findings provide a scientific basis for selecting ellipsoidal reduction strategies in engineering surveying practice and confirm the superiority of the generalized differential calculus method in constructing engineering ellipsoids.

In precision engineering surveying, the reduction of ground-observed distances to the reference ellipsoid results in a systematic shortening, denoted as $\:{\delta\:}_{1}$, which increases with the mean height * H* of the survey area. Calculations indicate that when * H* is approximately 159.3 m, the relative length distortion reaches about 25 mm/km, meeting the tolerance limits specified in general engineering surveying standards (Fig. 3a).

Conversely, projecting ellipsoidal lengths onto the Gauss plane introduces elongation. Except along the central meridian where no distortion occurs, the distortion $\:{\delta\:}_{2}$ increases quadratically with the y-coordinate of the survey point^28–30^. Theoretical analysis shows that when y reaches about 45.05 km, the relative distortion due to projection also amounts to 25 mm/km, which similarly complies with standard tolerance requirements (Fig. 3b).

In practical engineering applications, however, it is often challenging to simultaneously satisfy both H ≤ 159.3 m and y ≤ 45.05 km in order to control the total distortion within 25 mm/km (Fig. 4). Traditional approaches involve selecting an appropriate projection datum or shifting the central meridian for distortion compensation, or even combining both strategies^14,31,32^. Nevertheless, these methods exhibit certain limitations in practice: the procedures are cumbersome, and it remains difficult to guarantee computational accuracy across all regions. Therefore, there is a clear need to develop more systematic and adaptive reduction models.

The results indicate significant discrepancies after reducing the original electro-optical distance measurements to the mean elevation surface within the survey area^7,29,33^. Among the baselines analyzed, only YG01–YG02 and YG05–YG06 remained within the permissible tolerances, while the other five baselines exhibited clear exceedances. Notably, baseline YG03–YG04 showed the maximum length distortion of − 41.2 mm, substantially exceeding the specified tolerance of ± 12.6 mm for engineering surveys (Table 3; Fig. 9). These findings underscore the necessity and urgency of implementing length reduction corrections.

Therefore, this study focuses on the scaling method of reference ellipsoid parameters. It proposes scaling the semi-major axis by a certain proportion while keeping the flattening of the ellipsoid unchanged, so that the expanded ellipsoid more closely approximates the regional engineering average elevation surface within the target area^25,34,35^. This approach reduces projection distortion and enhances the accuracy and efficiency of local engineering surveys^3^. We systematically compared four mainstream ellipsoid expansion methods - mean curvature radius method, prime vertical curvature radius method, plane analytical method, and generalized differential method - in terms of their accuracy and applicability in reducing measured distances to a custom engineering ellipsoidal surface^3,11^. Through rigorous comparative analysis, this study aims to identify an optimal, minimally distorted engineering ellipsoid datum for specific survey areas, ensuring data quality and reliability throughout the entire workflow, from field observations to indoor mapping.

As traditional ellipsoid expansion methods, the mean curvature radius method and the prime vertical curvature radius method offer the advantage of computational simplicity^36^. However, in areas with significant topographic relief, these methods tend to produce systematic deviations in length reduction due to insufficient consideration of local curvature variations^35^. As a result, the semi-major axis derived from these methods shows notable differences compared to those obtained from the other two approaches (Tables 1 and 2; Figs. 6 and 8). Particularly at the edges of the survey area and in zones with sharp elevation changes, the residuals are largest, reflecting the inherent limitations of these methods in accurately characterizing local ellipsoidal properties^3,14^. Therefore, they are more suitable for preliminary or small-scale engineering projects where lower accuracy is acceptable.

Although the planar analytical method and the generalized differential method yield nearly identical adjustments to the semi-major axis after ellipsoid expansion - with differences only at the centimeter level (Table 2; Figs. 7 and 8) - they differ fundamentally in theoretical basis and applicability.

The planar analytical method projects observations based on the planar geometric relationship between the survey area center and individual measurement points^8^. It offers intuitive concepts that are easily understood in engineering practice and effectively controls projection distortion near the center of the survey area^27^. However, this method is essentially a simplified or approximate form of rigorous conformal mapping (such as Gaussian projection). When applied to large or irregularly shaped areas, it struggles to achieve globally minimized projection distortion, often resulting in excessive deformation at the edges of the survey area^10,20,24^.

This study builds upon previous research. Early engineering surveying textbooks and standards widely recommended the mean curvature radius method (M method) and the prime vertical curvature radius method (N method), emphasizing their advantage of computational simplicity^11,19^. Subsequent scholars progressively introduced the planar analytical method (P method), highlighting its significant value in controlling projection distortion and preserving geometric similarity^3,4^.

After applying the generalized differential method to establish the engineering ellipsoid and reduce the observed ground distances, the results demonstrate that the length distortions of all seven baselines in the study area are within the tolerance limits specified by the relevant engineering surveying standards. Among them, baseline YG07-YG08 exhibits the smallest distortion, merely 1.8 mm, well below its allowable tolerance of ± 8.5 mm. Meanwhile, the maximum distortion observed − 11.4 mm for baseline YG01-YG02 - also satisfies the corresponding tolerance requirement of ± 37.9 mm (Table 4; Fig. 9). These findings validate the effectiveness and superiority of the generalized differential method in controlling projection distortion.

Meanwhile, this study compares the lengths calculated by the four ellipsoid expansion methods with the electro-optical distance measurements. The results show that the length deformations obtained by the mean curvature radius method and the prime vertical radius method exceed the tolerance, while both the planar analytical method and the generalized differential method meet the tolerance requirements. Among them, the generalized differential method yields the smallest length deformation (Table 5).

This study also has several limitations. First, the experimental analysis was primarily conducted within a specific topographic and geographical context. Although sensitivity analyses were performed by varying the extent and elevation differences, the applicability of our conclusions under extreme topographic conditions - such as ultra-long linear projects or ultra-high-altitude regions - requires further validation. Second, while the generalized differential method demonstrates notable advantages in accuracy, its computational complexity and dependence on specialized software platforms have not been thoroughly examined. Restricted by field survey conditions and data access authority, multi-regional measured samples are currently unavailable. This limitation has been clarified in the manuscript, and multi-scenario extended verification will be regarded as a key direction for future research. In practical engineering applications, the precision gains achieved by this method may not justify the increased time and labor costs for smaller-scale projects.

Building upon the aforementioned research findings and existing limitations, future studies could be further advanced in the following aspects:

Constructing a Multi-Scenario Case Library and Evaluation System: Systematically collect data from typical test areas worldwide to assess the applicability of the generalized differential method under various geological conditions, climate zones, and latitudinal regions. Establish an evaluation index system for method applicability to promote its standardized and normative application.

Developing Intelligent Optimization Algorithms: Integrate artificial intelligence and high-performance computing technologies to develop rapid approximation algorithms or adaptive mesh optimization strategies for the generalized differential method. This aims to significantly reduce computational costs while maintaining accuracy, thereby enhancing its feasibility and efficiency in practical engineering applications.

Integrating Multi-Source Physical Information: Incorporate multi-source data such as gravity field models and ground-penetrating radar to develop an engineering ellipsoid model that integrates geometric and physical constraints. This will further improve the accuracy and physical interpretability of reduction results, particularly enhancing the model’s reliability and explanatory power in regions with significant gravity anomalies.

In summary, this study, through systematic empirical analysis, establishes the significant advantages of the generalized differential calculation method in constructing engineering ellipsoids, providing reliable theoretical and methodological support for high-precision engineering surveys. With the continuous advancement of related technologies and the deepening of engineering applications, the generalized differential method is expected to play a pivotal role in a broader range of surveying scenarios, driving engineering surveying technology toward higher precision, enhanced adaptability, and greater intelligence.

## Conclusion

This study selected seven baselines in a high-altitude area and performed precise measurements using the high-precision measurement robot TS60. The measured results were compared with two types of projected lengths: one reduced to the engineering projection plane at the mean elevation of 3437 m in the survey area, and the other projected onto an engineering ellipsoid constructed based on the generalized differential method and ellipsoidal expansion theory. The results demonstrate that the ellipsoidal expansion model established via the generalized differential method effectively controls projection length distortion, with the deformation meeting the tolerance limit of 25 mm/km specified by engineering survey standards. The proposed method is straightforward to implement and exhibits strong practicality and reliability, making it suitable for the accurate processing of projected lengths in engineering projects located in high-altitude regions.

The results indicate that when reducing the ground-surveyed length to the ellipsoidal surface, the length distortion manifests as shortening, and the magnitude of this distortion is positively correlated with the mean elevation of the survey area - higher elevations lead to greater deformation. To satisfy the length distortion tolerance of 25 mm/km stipulated by engineering survey specifications, the mean elevation of the area should not exceed 159.3 m. Conversely, when projecting the ellipsoidal length onto the Gauss plane, the length distortion appears as elongation, with its magnitude depending on the distance of the area from the central meridian - greater distances result in increased distortion. Under the same tolerance requirement, the mean abscissa of the survey area must be kept within 45.05 km. In regions characterized by both high elevation and significant distance from the central meridian, simultaneously meeting these conditions to comply with the standard becomes notably more challenging.

After evaluating the advantages and disadvantages of four ellipsoidal expansion methods, the generalized differential method was ultimately selected in this study to establish the engineering ellipsoid. To validate its effectiveness, the ground-surveyed lengths of seven baselines in the survey area were projected onto both this engineering ellipsoid and the local engineering projection plane, respectively. The results show that the length distortion generated by projection onto the engineering ellipsoid is significantly smaller than that onto the engineering projection plane, with the deformation fully satisfying the tolerance limit of 25 mm/km specified in engineering survey standards.

In summary, the generalized differential ellipsoidal expansion method effectively controls length distortion at its source by constructing an optimal local ellipsoid for a specific project area. This approach ensures the unification of the spatial datum and enhances data accuracy in high-precision surveying for large-scale or special terrain engineering projects, thereby providing a valuable reference for related research and practice.

## Data Availability

Data will be made available on reasonable request.

## References

[1] Vestol, O., Breili, K. & Taskjelle, T. Common adjustment of geoid and mean sea level with least squares collocation. *J. Geodesy*. **99** (5). 10.1007/s00190-025-01961-7 (2025).

[2] Tullu, A., Cho, Y. & Ko, S. Gaussian mixture model-based vector approach to real-time three-dimensional path planning in cluttered environment. *IEEE Access.* **13**, 8077–8091. 10.1109/ACCESS.2025.3527123 (2025).

[3] Lapaine, M. Gauss-Kruger projection as a double mapping. *Geodetski List.* **75** (2), 85–102 (2021).

[4] Guo, J. C., Shen, W. B. & Ning, J. S. Development of Lee’s exact method for Gauss-Kruger projection. *J. Geodesy*. **94** (6). 10.1007/s00190-020-01388-2 (2020).

[5] Hanief, I. et al. Graphene-based nanomaterials for strain detection in smart concrete. *Arab. J. Sci. Eng.* **51** (1), 503–540. 10.1007/s13369-025-10981-6 (2026).

[6] Yuan, B. et al. Dynamic behavior and deformation of calcareous sand under cyclic loading. *Soil Dyn. Earthq. Eng.* **199**, 109730. 10.1016/j.soildyn.2025.109730 (2025).

[7] Yang, W. X., Li, T. T., Wen, B. & Ren, Y. Correlation cluster analysis of slope safety monitoring data in reservoir areas. *PLOS ONE*. **20** (5). 10.1371/journal.pone.0324604 (2025). 10.1371/journal.pone.0324604 PMC12119109 40435450

[8] Long, F., Xia, X., Zhang, B. & Feng, Q. A Six-Degree-of-Freedom (6-DOF) Simultaneous Measurement Method Using Dual-Wavelength Laser Sources for Compensation of Air-Turbulence-Induced Beam Deviation. *Sensors* **25** (23). 10.3390/s25237122 (2025). 10.3390/s25237122 PMC12694237 41374493

[9] Yuan, B. et al. Effects of particle size on properties of engineering muck-based geopolymers: Optimization through sieving treatment. *Constr. Build. Mater.* **492**, 142967. 10.1016/j.conbuildmat.2025.142967 (2025).

[10] Chen, Y. et al. High-update-rate (25 kHz) laser ranging with random noise modulation for fast and precise absolute distance measurement. *Sensors* **25** (22). 10.3390/s25226985 (2025). 10.3390/s25226985 PMC12656084 41305192

[11] Cazabal-Valencia, L., Caballero-Morales, S. O., Martínez-Flores, J. L. & Gibaja-Romero, D. E. Capacitated Multi-Facility Location Problem on the Ellipsoid with Multi-Zone Restrictions. *Int. J. Comb. Optim. Probl. Inf.* **13** (3), 75–86 (2022).

[12] Fu, Y. G. et al. Development of an ellipsoid based chart datum around the China Seas. *Mar. Geodesy*. **48** (4), 452–475. 10.1080/01490419.2025.2479618 (2025).

[13] Eleiche, M. & Mansi, A. H. Exact and heuristic formulae to compute the geodetic height from the ellipse equation. *Geodesy Geodyn.* **15** (2), 150–155. 10.1016/j.geog.2023.05.007 (2024).

[14] Li, X. Y., Li, H. P., Liu, G. H. & Bian, S. F. Optimization of Complex Function Expansions for Gauss-Kruger Projections. *ISPRS Int. J. Geo-Information*. **11** (11). 10.3390/ijgi11110566 (2022).

[15] Li, P. F. et al. A target geolocation method using laser trilateration under ECEF frame with atmospheric refraction correction. *J. Spat. Sci.* **70** (2), 275–293. 10.1080/14498596.2024.2426459 (2025).

[16] Lapaine, M. A New Derivation of the Formula for the Length of a Loxodrome Arc on a Sphere Using Cylindrical Projections. *ISPRS Int. J. Geo-information*. **14** (4). 10.3390/ijgi14040137 (2025).

[17] Seitz, K., Heck, B. & Abd-Elmotaal, H. External gravitational field of a homogeneous ellipsoidal shell: a reference for testing gravity modelling software. *J. Geodesy*. **97** (6). 10.1007/s00190-023-01733-1 (2023).

[18] Quan, W., Zhang, J. J. & Liu, C. Improved chord method for the direct and inverse problems of the distances and azimuths on the reference ellipsoid. *Surv. Rev.* **55** (391), 361–368. 10.1080/00396265.2022.2108274 (2023).

[19] Chang, S. S. et al. Two algorithms of geodesic line length calculation considering elevation in eLoran systems. *IET Radar Sonar Navig.* **17** (10), 1469–1478. 10.1049/rsn2.12435 (2023).

[20] Kerkovits, K. Secant Cylinders Are Evil-A Case Study on the Standard Lines of the Universal Transverse Mercator and Universal Polar Stereographic Projections. *ISPRS Int. J. geo-information*. **13** (2). 10.3390/ijgi13020056 (2024).

[21] Gao, Z., Rong, X., Wang, W., Huang, B. & Liu, J. Q. TBM advanced geological prediction via ellipsoidal positioning velocity analysis. *Buildings* **14** (10). 10.3390/buildings14103126 (2024).

[22] El-Husseiny, A. Predicting Variations in P-Wave Velocity as a Function of Pressure in Carbonates: An Artificial Neural Network Approach Incorporating the Impact of Rock Properties. *Arab. J. Sci. Eng.* **51** (1), 255–271. 10.1007/s13369-025-11024-w (2026).

[23] Yuan, B. et al. Study on the interaction between pile and soil under lateral load in coral sand. *Geomech. Energy Environ.* **42**, 100674. 10.1016/j.gete.2025.100674 (2025).

[24] Zhang, Q. J., Cai, S. S. & Liang, X. L. Individual tree segmentation in occluded complex forest stands through ellipsoid directional searching and point compensation. *For. Ecosyst.* **11**10.1016/j.fecs.2024.100238 (2024).

[25] Yang, Y. F., Zhang, Y. F., Liu, Q., Lv, X. Q. & Huang, P. A rotational ellipsoid model for solid Earth tide with high precision. *Sci. Rep.* **14** (1). 10.1038/s41598-024-79898-8 (2024). 10.1038/s41598-024-79898-8 PMC11574028 39558007

[26] Wu, Y. & Qian, X. (eds) The Establishment of Engineering Coordinate System and Its Coordinate Transformation by Changing the Geometric Parameters of Ellipsoid. IOP Conference Series: Earth and Environmental Science; : IOP Publishing. (2021).

[27] Hwang, C., Kuo, C. Y., Shih, H. C., Huang, W. H. & Lan, W. H. Depth modernization by integrating mean sea surface model, ocean tide model, and precise ship positioning. *J. Geodesy*. **99** (3). 10.1007/s00190-025-01949-3 (2025).

[28] Yao, Z. G., Yu, T., He, J. Y. & Fang, Y. Experimental study on the effects of dynamic disturbance on Stick-Slip of structural planes in underground engineering. *Tunn. Undergr. Space Technol.* **165**10.1016/j.tust.2025.106866 (2025).

[29] Yang, W. X., Li, T. T., Wen, B. & Miao, Z. X. Correlation analysis and comprehensive evaluation of dam safety monitoring at Silin hydropower station. *Sci. Rep.* **15** (1). 10.1038/s41598-025-15094-6 (2025). 10.1038/s41598-025-15094-6 PMC12343999 40796943

[30] Prijatna, K., Lestari, R., Bramanto, B., Pahlevi, A. M. & Wijaya, D. D. Assessing the impact of methodological differences on geoid model performance. *Geographia Technica*. **19** (2), 124–138. 10.21163/GT_2024.192.09 (2024).

[31] Guo, R. H., Cui, H. H., Jiang, T., Wei, D. C. & Cheng, X. S. A pose optimization method for field measurement accuracy improvement of the visual Tracking-Based measurement system. *Measurement* **253**10.1016/j.measurement.2025.117663 (2025).

[32] Rollins, C. M. & Meyer, T. H. Conformal mapping at elevation with implications for low-distortion projections. *Surv. Rev.* **56** (399), 606–619. 10.1080/00396265.2024.2348851 (2024).

[33] Dhabaan, H. A. et al. Experimental Investigation of the Shear Performance of Concrete Beams Reinforced with BFRP Bars. *Arab. J. Sci. Eng.*10.1007/s13369-026-11186-1 (2026).

[34] Fang, R. K. et al. Deformation and failure mechanisms of slopes in the Loess Plateau with structural planes under engineering excavation. *Geomorphology* **486**10.1016/j.geomorph.2025.109888 (2025).

[35] González, C. J., Torres, J. R., Haro, S., Gómez-Enri, J. & Alvarez, O. High-resolution characterization of intertidal areas and lowest astronomical tidal surface by use of Sentinel-2 multispectral imagery and hydrodynamic modeling: Case-study in Cadiz Bay (Spain). *Sci. Total Environ.* **861**10.1016/j.scitotenv.2022.160620 (2023). 10.1016/j.scitotenv.2022.160620 36464044

[36] Filmer, M. S. et al. Connecting Land and Sea Vertical Datums: A Data Evaluation for Developing Australia’s AUSHYDROID Model. *Mar. Geodesy*. **47** (3), 183–213. 10.1080/01490419.2024.2305898 (2024).

